# Patterns and correlates of physical activity in adult Norwegians: a forecasted evolution up to 2025 based on machine learning approach

**DOI:** 10.1186/s12889-018-5854-2

**Published:** 2018-07-25

**Authors:** Alessio Rossi, Giovanna Calogiuri

**Affiliations:** 1 Department of Public Health, Faculty of Social and Health Sciences, Inland Norway University of Applied Sciences, Hamarveien 112, 2411 Elverum, Norway; 2 Department of Computer Science, University of Pisa, Largo Bruno Pontecorvo, 3, 56127 Pisa, PI Italy

**Keywords:** Physical activity patters, Physical activity correlates, Machine learning, Prediction

## Abstract

**Background:**

As other westerns countries, a large portion of Norwegians do not meet the minimum recommendations for weekly physical activity (PA). One of the primary targets of the WHO’s *Global action plan for the prevention and control of noncommunicable diseases* is to reduce insufficient PA by 10% within 2025. In order to effectively increase the PA levels in the population, an in-depth understanding of PA habits within different sub-groups is therefore vital. Using a machine learning (ML) approach, the aim of this study was to investigate patterns and correlates of PA in adult Norwegians, as well as to construct a predictive model of future PA.

**Methods:**

Data were retrieved from the Norsk Monitor survey, which consists of about 3000 items on individual characteristics and sociocultural factors. The dataset contained information about 52,477 adult Norwegians, collected between 1985 and 2013. Past patterns and changes of three PA components (*Frequency*, *Duration*, and *Intensity*) were initially assessed using a series of ANOVAs. A Conditional Mutual Information Maximization Method and a recursive feature elimination with cross-validation were then used to examine the factors associated with such patterns and changes. Finally, the future evolution of the three PA components up to 2025 was predicted using an autoregressive model.

**Results:**

In line with previous literature, the analysis of the PA patterns showed a progressive increment of the PA *Frequency* (which was greater in women), while the PA *Duration* and *Intensity* (which were in general higher among men) resulted fairly stable. The PA correlates identified by the ML analysis, which include men and women of different age groups, are presented and discussed. The autoregressive model predicted a general increment of the PA *Frequency* and PA *Intensity* by 2025, while the PA *Duration* is predicted to reduce. Different patterns emerged among the different sub-groups, overall suggesting smaller increments of PA in men and older individuals, as compared to women and younger individuals.

**Conclusions:**

The findings of this study can inform public health efforts that aim at increasing PA levels in specific target groups. The ML approach is proposed as a useful tool in public health monitoring and assurance.

## Background

The World Health Organization (WHO) recommends that adults engage in moderate-intensity aerobic physical activity (PA) for at least 150 min a week, or in vigorous-intensity aerobic PA for at least 75 min a week, or an equivalent combination of moderate- and vigorous- intensity [1]. From these recommendations, three components, namely frequency, duration and intensity, emerge as central in order to estimate the extent to which a person meets the PA recommendations. *Frequency* refers to how often a person engages in PA [1]. Health institutions such as the Norwegian Directorate of Health (NDH) recommend individuals to engage in PA bouts as often as possible in order to avoid the deleterious effects of prolonged exposure to sedentary behaviours [2]. *Duration* refers to the length of time in which a given activity is performed. According to the WHO’s recommendations, aerobic PA bouts should have a minimum duration of 10-min [1], while the NDH encourages adults to engage in at least 30-min of PA every day [3]. Finally, *Intensity* refers to the rate at which a given activity is performed. Generally, the WHO’s and NDH’s guidelines refer to two levels of PA *Intensity*: ‘moderate’ and ‘vigorous’. In terms of a person’s *perceived* exertion, moderate-intensity PA refers to any activity that, on a scale of 0–10, would be rated 5–6, whereas vigorous-intensity PA would be rated 7 or higher [1].

An insufficient PA is known to be one of the leading risk factors for mortality worldwide. Nevertheless, a large portion of the population still does not meet the minimum recommended levels for PA [4]. To increment the PA levels in the population is therefore a public health priority. Reducing insufficient PA by 10% within 2025 is, in fact, the third target of WHO’s *Global action plan for the prevention and control of noncommunicable diseases 2013–2010* [4]*.* Monitoring the PA patterns in the population as well as understanding the individual and environmental factors that are associated with them are important steps in developing effective policies and initiatives aiming to encourage more active lifestyles. Furthermore, predictions of future changes of PA habits in different population’s sub-groups is important in order to evaluate the effectiveness of public health efforts to increase the population’s PA, as well as the expected burden due to insufficient PA.

In modern societies, the PA habits of the population have been changing alongside with changes of a number of socioeconomic and environmental factors, such as the increasing prevalence of sedentary working conditions, changes in preferences for leisure time activities, and an increment in the employment of motorized means of transportation. National surveys show that adult Norwegians are, in general, relatively active: self-reported measurements indicate that about 65% of adult Norwegian meet the WHO’s minimum recommendations for PA, although recent studies based on accelerometer assessments revealed that only one out of three Norwegians actually meet the PA recommendations [5, 6]. However, a rise in obesity and sedentary behaviours across all age groups have been detected [7]. Moreover, even though Norwegians appear to have increased their PA levels the from 1985 to 2011 [8], it has been argued that this increment is not enough to compensate the growing time spent in sedentary behaviours at work and during leisure time [9]. Therefore, although several studies of PA patterns in the Norwegian population have been conducted, in light of the problematics highlighted above, there is still a need for an in-depth understanding of this phenomenon.

In recent years, the use of Machine Learning (ML) approaches has been proposed as a useful tool to investigate the behavioural, social, and environmental aspects affecting the population’s health [10]. ML is a process that enables computer systems to progressively improve performance on a specific task without being explicitly programmed, and it can be used for data analysis purposes in order to identify patterns within a high-volume dataset and to make predictions based on these patterns [11]. This approach has been previously applied to study health outcomes based on routinely collected data in different populations. For example, ML has been used to develop a predictive model to prevent injuries of soccer players based on GPS measurements collected during training sessions [12]. ML techniques have also been used to predict the risk of death based on patient care records and information from population surveys [13]. To the best of our knowledge, however, ML analysis has not been applied yet to the study of populations’ PA based on periodic national surveys. Studies based on information collected by mobiles applications [5] or exercise apparatus [14], have been carried out to investigate PA patterns at large-scale (i.e. at European and World-wide level). However, compared with these studies that primarily focus on PA patterns, national surveys can provide more information about the characteristics of individuals and their living environments. Not only can this help researchers to depict PA patterns within a population, it can also help to study correlates of such PA patterns in order to develop predictive models.

Hence, the purpose of this study was to investigate patterns and correlates of PA (expressed in terms of frequency, duration and intensity) in the Norwegian population using the data collected by a large national survey which started in 1985. Using ML techniques, we investigated which factors can be associated with the evolution of PA patterns throughout the past three decades in a sample of adult Norwegians, including individuals’ beliefs and values, preferences for particular types and locations of PA, and perceived barriers. Finally, we developed a predictive model of future PA patterns in the Norwegian population to make evaluations about the extent to which Norway is expected to meet the WHO’s goal of reducing insufficient PA within 2025.

## Methods

### Participants and data

The data for this study contained information about 52,477 Norwegians who participated to a series of waves of the Norsk Monitor survey between 1985 and 2013. Norsk Monitor is a large cross sectional survey administered biannually since 1985, which consists of about 3000 items covering topics such as media usage, social policy issues, consumer behaviour, eating habits and political views. Since many of the questions are asked repeatedly through the years, Norsk Monitor is frequently used in media and social sciences to elucidate attitude changes over time. The survey has shown high reliability and validity [15–17].

### Dependent features

**Fig. 1:**
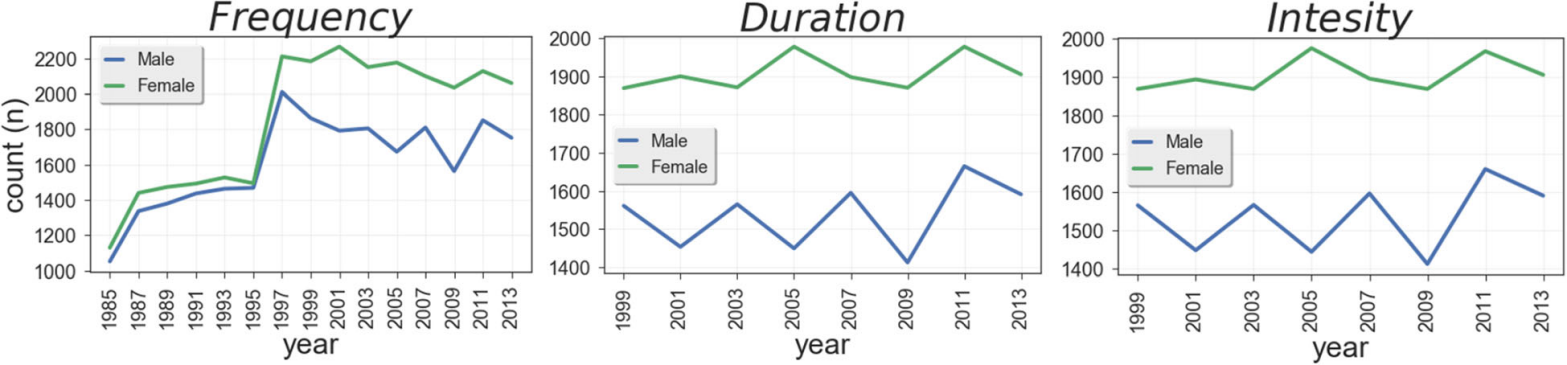
Number of survey respondents for each year by gender. This figure shows the number of survey respondents recorded for each PA component in each year by gender

Three items relative to three major PA components were used as independent features for this particular study: i) *Frequency*; ii) *Duration*; iii) *Intensity* (see the introduction for an explanation of these PA components). Each of these PA components was assessed through a closed-ended multiple-choice item (Appendix 1): eight options were provided for *PA Frequency* (1 = “Never”; 8= “Once or more every day”), while six options were provided for *PA* D*uration* (1= “Less than 15 minutes”; 6= “More than 1.5 hours”) and PA *Intensity* (1= “I don’t feel any change in my breath or body-heat”; 6= “I reach maximum exhaustion”). Because of the way these measures are constructed in the Norsk Monitor survey, it was impossible to re-code them in a way able to allow us to determine the prevalence of respondents meeting or not meeting the WHO’s recommendations for weekly PA. Moreover, PA *Frequency* was recorded since 1985, while PA *Duration* and *Intensity* were added starting from 1999. The response to *Frequency* for the three PA components throughout all survey waves available are shown in Fig. 1.

### Independent features

Because of known differences of PA patterns between genders and among age groups [6], all analyses were stratified by these variables. Age, which was available in the Norsk Monitor survey as a continuous variable, was categorized in four groups: < 25 years old (yo), 25–44 yo, 45–64 yo, and ≥ 65 yo All the other items in the dataset were used as independent features. Appendix 2 provides the most relevant items (as based on the Gini Coefficient) detected by the analysis described in the following section. The surveys containing answer options such as *‘I don’t know’, ‘I’m not sure’* and *‘I cannot answer’* in the independent features, were deleted from the dataset by a case-wise deletion approach. Only 0.65 and 0.73% of the answers was deleted for females and males, respectively. Due to the low rate of missing data answers, the analyses were not relevantly affected.

### Statistical analysis

#### Preliminary analysis

Before performing the ML analyses, a set of ‘classical’ statistical tests were performed in order to i) examine possible interdependence among the different dependent features, ii) detect possible interactions between gender and age, and iii) examine patterns and trends in the dataset. These preliminary analyses were necessary in order to better plan the subsequent ML analyses, as well as to better interpret the final findings. Any possible correlation between the dependent features (i.e., PA *Frequency*, PA *Duration* and PA *Intensity*) was assessed by Spearman’s rank correlation coefficient [18] using the original 6- or 8-point component scales. The correlations were repeated for each gender and age group, separately. A series of two-ways analyses of variance (ANOVA) [19] were performed to detect possible differences between genders and among age groups as well as their possible interactions. A two-ways ANOVA was also performed in order to detect possible interactions of gender or age group by survey wave. All ANOVAs were performed for each PA component separately. Before these analyses, the normality of the data distribution and the sphericity assumptions were assessed by Shapiro-Wilk’s Normality Test and Mauchly’s Test, respectively (the assumptions of normal data distribution and Sphericity were met). Pairwise comparisons for the ANOVAs were performed using Tukey’s Honest Significant Difference Post Hoc Test (HSD). Finally, an autocorrelation analysis [20] was performed in order to detect repeating pattern in a time series as a function of the time lag between them. *Frequency* showed a significant autocorrelation at lag 1 and 2 (*r* > 0.6) for both men and women in all age groups, indicating that the time series had a high degree of autocorrelation between adjacent and near-adjacent observations. In order to avoid autocorrelation effects in the following ANOVA analyses, a correction on the year factor was applied. Differently, no significant autocorrelation was found in *Duration* and *Intensity.* All statistical analyses were performed using Python version 2.6., while significance level was set at *p* < 0.05.

#### Machine learning approach

##### Construction of training dataset

*Given* a feature set *S*, the training dataset T_*S*_ for the learning task was built following a two-steps procedure:

1. For every respondents *i* we built a feature vector **m**_***i***_ = (*h*_*1*_*,…,h*_*k*_) where *hj ∈ S, (j = 1,…,k)* is a survey answer and *k* = |*S*| is the number of items considered. All the feature vectors compose matrix *F*_*S*_ *= (****m***_***1***_*,…****m***_***n***_*)*, where *n* is the number of individual survey in our dataset (*n* = 52,477).
2. Every feature vector **m**_***i***_ was associated to three labels *c*_*i*_ ∈ {0, 1, 2} (i.e., the item answers were grouped into 3 ordinal classes. For more detail see Appendix 1). Matrix *F*_*S*_ was hence associated to three vectors of labels *c* = (*c*_*1*_*,…,c*_*3*_) (i.e., one for each independent feature). The training dataset for the learning task was *T*_*S*_ = (*F*_*S*_*,c*). Table 1 shows an example of the dataset.

**Table 1:** Structure of the training dataset

	h_1_	h_2_	…	h_k_	C_1_	C_2_	C_3_
s_1_	2	3	…	2	2	1	3
s_2_	1	1	…	2	2	2	1
s_3_	3	2	…	1	1	1	3
⁞	⁞	⁞	⁞	⁞	⁞	⁞	⁞
s_*n*_	1	2	…	2	3	3	2

Each example *s* describes a vector of items *m*_*i*_ consisting of k features (*h*_*k*_*)* and three labels c ∈ {1, 2, 3}. The light black reflects the vectors of features; otherwise, the dark black reflects the labels. Label 1, 2 and 3 reflects the item *Frequency*, *Duration* and *Intensity*

Eight different datasets were constructed for both men and women in the different age groups and the classifiers were built for each of the three labels. Therefore, 24 classifier performances have been provided in this study.

##### Experiments

A feature selection process based on two steps allowed us to reduce the number of predictive features into a manageable number. First of all, a feature selection by Conditional Mutual Information Maximization Method (CMIM)^1^ [21] was applied. This algorithm allowed the selection of a subset of features that carries as much information as possible minimizing the entropy function *Ĥ(y|(h*_*1*_*,…,h*_*k*_*)* without setting an initial threshold for the number of features to retain. Secondly, a recursive feature elimination with cross-validation (RFECV)^2^ [22] was applied using the same algorithm for the future classification task. These processes were performed in order to reduce the dimensionality of the feature space (i.e., survey respondents) and the risk of overfitting, allowing a more manageable interpretation of the ML models thanks to the lower number of features. The importance of the features was assessed by a normalized Gini Coefficient. The feature selection process was performed for every age group and for both genders, as well as for each label. On the dataset derived from the feature selection, we trained two different classifiers: Ordinal Regression (Ordinal)^3^ and Random Forest Classifier (RF).^4^

The classifiers were validated with a 3-fold stratified cross-validation strategy. The real dataset was split into three folds where 90% of the dataset was used as training set, while the remaining 10% of the target values was used as test set. Each fold was made by preserving the percentage of samples for each class. Thus, each sample in the dataset was tested once, using a model that was not fitted with that sample. The goodness of the classifiers was assessed by Precision, Recall and F1-score (f1). Precision indicates the fraction of examples that the classifier correctly classifies over the number of all examples that itassigns to that class. Recall indicates the ratio of examples of a given class correctly classified by the classifier, while F1-score is the harmonic mean of precision and recall. In particular, ‘recall’ refers to the percentage of cases that were correctly labelled, while ‘precision’ indicates the trustworthiness of the classifier’s predictions: the higher the precision, the more a classifier’s predictions are reliable. Moreover, in order to assess the validity of the classifiers we compared our predictive models with two baselines. Baseline *B*_*1*_ randomly assigned a class to an example by respecting the distribution of classes, while Baseline *B*_*2*_ always assigned the majority class.

In order to forecast the PA components up to 2025, an autoregressive model^5^ [23] was created, which predicted the evolution of the three different PA components based on a weighted sum of the previous 6 years values reflecting the secular trend. More specifically, for both genders, a time series of the means of the different PA components in each survey wave was created. For each time series, the model was trained on n-3 elements of the time series (i.e., data recorded until 2007) and tested on the remaining three survey waves (i.e., 2009, 2011 and 2013 surveys). The accuracy of our model was assessed by computing the mean squared error (MSE) between the observed values and the predicted ones. To test the stationarity null hypothesis of autoregressive model, an F-test was performed. Finally, the model created was used to forecast the future changes for each of the three PA components, separately for both genders and the different age groups.

## Results

### Physical activity patterns from 1985 to 2013

Table 2 shows the findings of the correlation analysis among the different PA components from 1999 to 2013, showing weak positive linear relationships among the independent features. Even though these relationships were statistically significant, the small correlation coefficient suggests that these components are largely independent from each other.

**Table 2:** Correlation between PA characteristics

		Male		Female	
		Frequency	Duration	Frequency	Duration
< 25	Duration	0.22	–	0.28	–
	Intensity	0.27	0.37	0.23	0.34
25–44	Duration	0.15	–	0.15	–
	Intensity	0.24	0.30	0.18	0.32
45–64	Duration	0.12	–	0.13	
	Intensity	0.22	0.30	0.12	0.29
> 65	Duration	0.14	–	0.16	–
	Intensity	0.17	0.32	0.11	0.32

Spearman’s rank correlation coefficient from 1999 to 2013 based on the original 6- or 8-point component scales between the independent features (*Frequency*, *Duration* and *Intensity*) in both male and females and in the four age groups (i.e., < 25, 25–44, 45–64, > 64 years old)

**Fig. 2:**
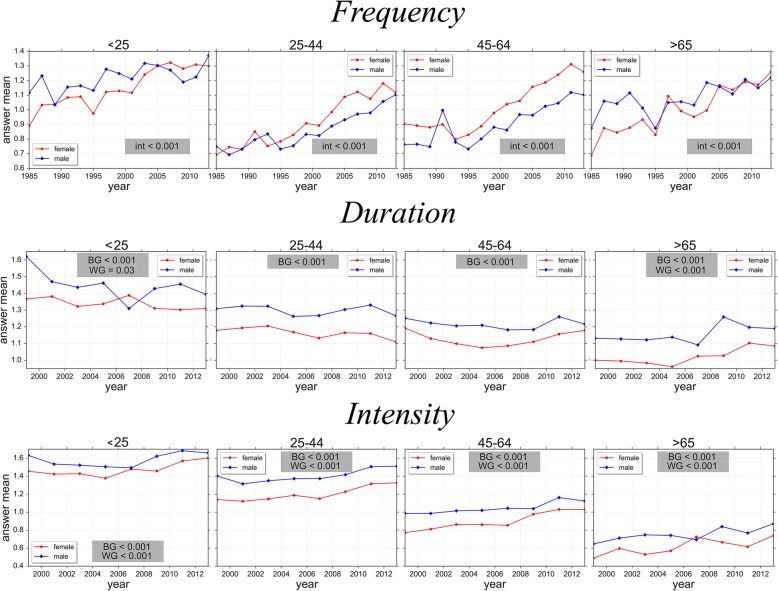
PA differences among age groups. This figure shows the differences between males and females among age groups computed by using Tukey’s HSD post-hoc test. The values provided in this figure are based on the original 6- or 8-point component scales. * refers to the statistical difference vs < 25 age group; *&* refers to the statistical difference vs 25–44 age group; *$* refers to the statistical difference vs 45–64 age group; *+* refers to the difference vs > 65 age group

Mean values of the three PA components for men and women in the different age groups are presented in fig. 2. No statistical interactions of gender among age groups were found for any of the PA components. However, an univariate test showed statistical differences among age groups for PA *Frequency* (F_(3,631)_ = 194.10; *p* < 0.001) and *Intensity* (F_(3,553)_ = 68.21; *p* < 0.001) in men. In particular, men in the age groups < 25 yo and > 65 yo reported higher PA *Frequency* compared to middle age men, while PA *Intensity* was lower in older men than in younger men. As for women, significant differences for PA *Duration* (F_(3,630)_ = 78.01; *p* < 0.001) and *Intensity* (F_(3,551)_ = 73.52; *p* < 0.001) were also detected. In particular, younger women reported higher PA *Duration* as compared as all other age groups, while PA *Intensity* was higher in younger women that in older ones.

**Fig. 3:**
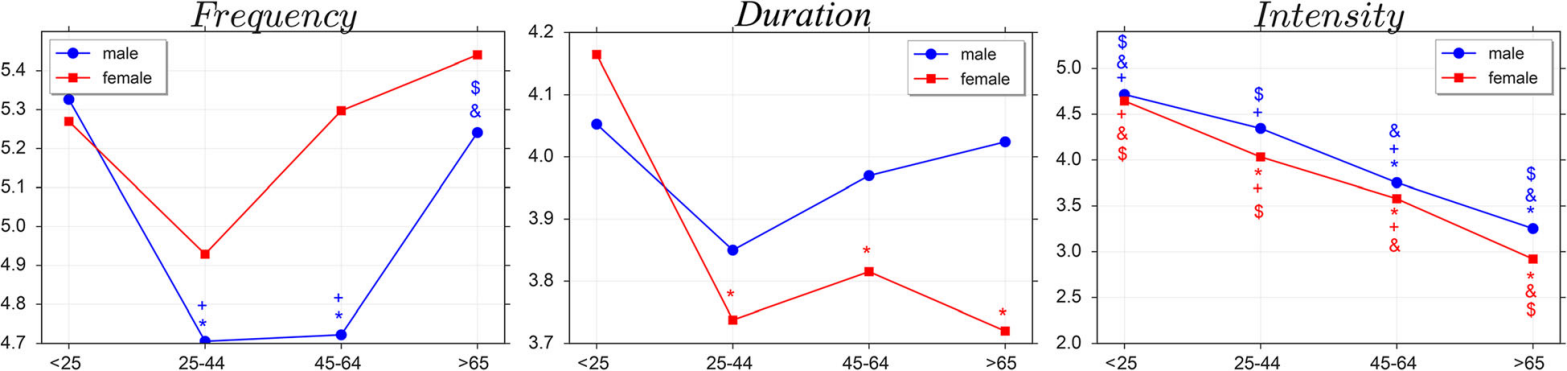
PA differences among years. In this figure we present: i) the evolutions of the *Frequency* in both males and females from 1985 to 2013; ii) the evolutions of *Duration* a week in both males and females from 1999 to 2013; iii) the Evolutions of *Intensity* in both males and females from 1999 to 2013. *Int, BG* and *WG* refer to the *p*-value of Interaction, Between Groups difference and Within Groups difference, respectively. The means for the different PA components are grouped in 3 ordinal classes

Mean values of the three PA components for men and women in the different age groups throughout all survey waves are presented in fig. 3. Significant interactions of gender over the years were found for PA *Frequency* in all age groups, with women showing a greater increase of PA *Frequency* from 1985 to 2013 than men. Noticeably, while in the earliest decade of the survey men reported in average higher PA *Frequency* than women, in the last decade women achieved and override the PA *Frequency* of men. No significant interactions of gender by survey wave was found for PA *Duration* and *Intensity* for any of the age groups, although men reported greater PA components than women.

### Correlates of the PA components until 2013

Table 3 shows the performance of the classifiers built on the base of the features selected in each dataset. The Ordinal regression model was chosen to present our findings since it showed the highest performance in all the 24 datasets (F1_*Ordinal*_ = 0.61 ± 0.13) compared to RF (F1_*RF*_ = 0.53 ± 0.07). This model also showed a higher performance when compared to the two baselines (F1_*B1*_ = 0.45 ± 0.08; F1_*B2*_ = 0.40 ± 0.10). The Ordinal Regression Model showed a high precision and recall compared to RF and the two baselines, indicating a high level of accuracy in classifying the respondents into different PA classes based on individual characteristics (see Table 3). Table 4 shows the features (for the sake of simplicity, only the five most relevant features, as based on the Gini Coefficient) selected in each of the 24 datasets. The sign of the coefficients provided in Table 4 reflects the direction of the relationship between the independent feature and the label (i.e., PA components): positive coefficient indicates that the higher is the independent feature, the higher is the PA label value, whereas negative coefficient indicates that the higher is the independent feature, the lower is the PA label value. The most important patterns emerged from these analyses are summarized below.

**Table 3:** Classification performances

			< 25			25–44			45–64			> 65		
			Prec	Rec	F1	Prec	Rec	F1	Prec	Rec	F1	Prec	Rec	F1
Frequency	Male	Ord	0.85	0.82	0.82	0.57	0.54	0.53	0.55	0.55	0.55	0.93	0.92	0.92
		RF	0.54	0.53	0.53	0.49	0.48	0.49	0.47	0.57	0.51	0.46	0.55	0.5
		B1	0.46	0.45	0.46	0.38	0.37	0.37	0.4	0.4	0.4	0.42	0.41	0.41
		B2	0.22	0.46	0.29	0.19	0.44	0.27	0.18	0.43	0.26	0.21	0.46	0.29
	Female	Ord	0.71	0.70	0.70	0.69	0.65	0.67	0.82	0.77	0.79	0.93	0.92	0.92
		RF	0.62	0.67	0.6	0.51	0.63	0.54	0.75	0.77	0.71	0.57	0.66	0.59
		B1	0.52	0.54	0.53	0.44	0.44	0.44	0.59	0.57	0.58	0.49	0.48	0.48
		B2	0.45	0.67	0.54	0.4	0.63	0.49	0.57	0.76	0.65	0.38	0.62	0.48
Duration	Male	Ord	0.94	0.95	0.94	0.54	0.61	0.54	0.55	0.57	0.56	0.80	0.77	0.76
		RF	0.67	0.72	0.69	0.54	0.61	0.54	0.55	0.57	0.55	0.44	0.5	0.47
		B1	0.55	0.56	0.7	0.42	0.42	0.42	0.34	0.35	0.35	0.42	0.42	0.42
		B2	0.41	0.64	0.5	0.33	0.57	0.42	0.25	0.5	0.33	0.22	0.47	0.3
	Female	Ord	0.72	0.71	0.72	0.63	0.69	0.65	0.49	0.58	0.5	0.81	0.78	0.76
		RF	0.72	0.61	0.49	0.46	0.68	0.55	0.47	0.51	0.49	0.38	0.62	0.48
		B1	0.53	0.52	0.52	0.53	0.54	0.53	0.43	0.41	0.42	0.44	0.48	0.46
		B2	0.33	0.57	0.42	0.46	0.68	0.54	0.33	0.58	0.42	0.38	0.62	0.47
Intensity	Male	Ord	0.61	0.70	0.63	0.78	0.77	0.78	0.51	0.48	0.49	0.86	0.81	0.82
		RF	0.62	0.67	0.65	0.53	0.56	0.53	0.38	0.51	0.42	0.45	0.55	0.47
		B1	0.52	0.54	0.53	0.48	0.47	0.47	0.39	0.4	0.4	0.39	0.39	0.39
		B2	0.45	0.67	0.54	0.33	0.57	0.42	0.23	0.48	0.31	0.26	0.51	0.34
	Female	Ord	0.67	0.71	0.69	0.59	0.65	0.62	0.49	0.51	0.53	0.80	0.72	0.7
		RF	0.58	0.66	0.59	0.61	0.62	0.61	0.44	0.51	0.5	0.41	0.48	0.44
		B1	0.49	0.49	0.49	0.48	0.48	0.48	0.41	0.42	0.42	0.34	0.34	0.34
		B2	0.46	0.68	0.55	0.24	0.49	0.31	0.27	0.52	0.36	0.23	0.48	0.31

Models metrics of PA *Frequency*, *Duration* and *Intensity* in all the age groups and in both males and females. Prec, Rec and F1 refer to precision, recall and f1-score, respectively. In the table, the models with higher F1 score are highlighted

**Table 4:** Individual characteristics and sociocultural factors associated with the respondents’ PA

		Male		Female	
		Feature	Coef	Feature	Coef
Frequency	< 25	PA habit - Strength exercise	0.97	Values - Healthy life	0.71
		Values – Debts	0.78	PA Habit - Handball	0.50
		PA Facilities - Sport hall	0.77	PA Facilities - Illuminated track	0.49
		PA Motive – Challenge	0.73		
		Climate Change	−0.70		
	25–44	Values - Healthy life	0.78	Values - Healthy life	0.98
		PA Facilities - Illuminated track	0.62		
		PA Facilities - Fitness Centre	0.49		
	45–64	PA Facilities - Walking trial	0.95	PA Facilities - Walking trial	0.60
		Values - Healthy life	0.77	PA Habit - Shooting	0.58
				PA barriers - Lack of enjoyment	−0.52
	> 65	Environmental behaviours - Active Transport	0.77	Duration	0.53
		PA Intensity	0.61	Environmental behaviours - Active Transport	−0.51
		PA Barriers - Lack of time	−0.58	Values – Strikes	0.49
		Comfort with divergences	−0.57	Close relationships with neighbours	−0.48
		Education field	0.55	Values - Healthy life	−0.44
Duration	< 25	PA Intensity	0.94	PA Habits – Sailings	0.75
		Values – Honesty	0.76		
		Childhood in a farm	0.73		
		Health benefits – Snus	−0.58		
		Values - Brands quality	0.55		
	25–44	PA Facilities- Walking trail	0.68	PA Facilities- Walking trail	0.57
	45–64	PA Facilities- Walking trail	0.66	PA Habit – Cycling	0.47
	> 65	PA Habit – Cycling	−0.62	PA Facilities - Outdoor area	0.54
		Language identity	−0.58	Values - Mothers with disabilities	0.53
		PA Facilities - Track and field stadium	0.52	Values - Healthy food	−0.52
		Religion inquiry	−0.50	PA Habits – Hiking	0.52
		Environmental organizations	0.49	Values - Personal liberty	0.49
Intensity	< 25	Disagreements with neighbour’s	−1.00	PA facilities - Fitness Centre	0.67
		PA Facilities - Walking trial	0.91	PA Habits – Hiking	0.53
		National pride	−0.89	PA Facilities – Motorsport	0.53
		Values - Children obedience	−0.87	PA Habits – Jogging	0.53
		Values - Countryside life	−0.86	Attitude to State Church	0.48
	25–44	View on social security	1.00	PA facilities - Fitness Centre	1.00
		PA Frequency	0.73		
		PA Motive – Appearance	0.71		
		Work field	0.69		
		Values - Economic equality	−0.58		
	45–64	PA Motive - Health benefits	0.97	PA Motive - Health benefits	1.00
	> 65	PA Habit – Cycling	0.72	Values – Gambling	−0.41
		Environmental concerns	−0.52	PA Frequency	0.40
		Values - Brands name	0.51	View on children number	0.40
		Religion identity – Christianity	−0.50	View on pension & holidays	−0.39
		PA Frequency	0.50	Values – Marriage	−0.39

The coefficient indicates the importance for the feature (as computed by Gini Coefficient) in predicting the different PA components F*requency*, *Duration* and *Intensity* in all the age groups (only five features with highest coefficient are shown)

#### Correlates of PA frequency

‘Healthy lifestyle’ was recurrently identified as a relevant feature predicting PA *Frequency*, with higher ratings of this feature predicting higher levels of PA *Frequency*. In addition, other features in the categories ‘PA habits’ and ‘PA facilities’ were also recurrently identified as relevant features predicting high levels of PA *Frequency*, though with different patterns between genders and among the different age groups. In particular, the features relative to engaging in indoor PA (i.e. strength exercise and playing handball) and using indoor PA facilities (i.e. sport halls and fitness centres) were more relevant in the younger age groups, whereas in older age groups the features relative to engaging in outdoor PA and using outdoor facilities (i.e. illuminated tracks and walking trails) were the most relevant. The other features predicting high PA *Frequency* were related to motives/barriers (e.g., seeking challenge; not perceiving enjoyment as a barrier) and to different values/views (e.g., believing that climate changes are not predominantly man-made; not engaging in active transport as an actions taken to preserve the environment).

#### Correlates of PA duration

Features in the category ‘PA habits’ and, especially, ‘PA facilities’ (often related to outdoor facilities, such as walking trails) were recurrently identified as relevant features predicting high levels of PA *Duration*, though with different patterns between genders and across the different age groups. Other features relative to values and health beliefs also emerged, especially in the youngest and oldest age groups. For instance, higher PA *Duration* was associated with higher PA *Intensity*, in < 25 yo men who have grown up in a farm and who assigned greater importance to values such as ‘Honesty’ and ‘Brands name’, and who perceive ‘Snus’ as having a negative impact on health. In men of various age groups, higher PA *Duration* was associated with supporting environmental organizations and using outdoor PA facilities (‘walking trails’ and ‘Track and field’), while lower PA *Duration* was associated with engaging in cycling, resorting to religion to address existential questions, and identifying with *Nynorsk* as a primary language (the latter probably a proxy of geographical distribution, as Nynorsk language is prevalent in rural areas of western Norway). Features relative to outdoor PA habits and places (i.e. engaging in sailing, cycling and hiking, and using walking trails and outdoor areas) emerged as particularly relevant predictors of high PA *Duration* among women in all age groups.

#### Correlates of PA intensity

In general, relevant features positively associated with high PA *Intensity* were related to PA habits (e.g. engaging in hiking and cycling in younger women and older men, respectively), PA places (e.g. using walking trails and fitness centres in younger men and all women but the oldest age group), and instrumental/extrinsic motives for PA (e.g. exercising for appearance and health benefits in middle-aged men). Having high PA *Frequency* was also recurrently associated with high PA *Intensity* (specifically, in 25–44 yo men as well as in > 65 yo men and women). On the other hand, several features depicting views and values on different issues were also identified as relevant predictors of PA *Intensity*, with unclear patterns characterizing different sub-groups of the sample.

### Forecasting PA changes

Table 5 shows the outcomes of the autoregressive model predicting the future changes of the three PA components up to 2025 based on the secular trend detected upon 2013. According to this model, men and women in all the age groups are expected to increase their PA *Frequency* of about 14.65 ± 6.11% (MSE = 0.004 ± 0.001; F_(1,6)_ ≥ 3.6e + 6, *p* < 0.001) and 41.10 ± 23.12% (MSE: 0.007 ± 0.002; F_(1,6)_ ≥ 3.6e + 9, *p* < 0.001) from 2013 to 2025, respectively. All the age groups in both males and females increased their PA *Frequency* from at least once every two weeks (< 1.02 arbitrary unit) in 1985 to once a week in 2013 (1.10–1.27 arbitrary unit), and will reach in mean more than once a week in 2025 (1.22–2.07 arbitrary unit). Moreover, the model predicts an increment in PA *Intensity* of about 7.98 ± 30.10% (MSE = 0.004 ± 0.002; F_(1,6)_ ≥ 3.6e + 12, *p* < 0.001) and 6.19 ± 29.46% (MSE = 0.003 ± 0.003; F_(1,6)_ ≥ 3.6e + 5, *p* < 0.001) from 2013 to 2025, in men and women respectively. To the contrary, the model predicts a reduction of PA *Duration* of about 14.78 ± 14.16% (MSE = 0.005 ± 0.002; F_(1,6)_ ≥ 3.6e + 8, *p* < 0.001) and 1.89 ± 5.08% (MSE = 0.007 ± 0.003; F_(1,6)_ ≥ 3.6e + 7, *p* < 0.001), in men and women respectively.

**Table 5:** PA components prediction

		Frequency			Duration			Intensity		
		1985	2013	2025	1999	2013	2025	1999	2013	2025
*Male*	*< 25*	1.02	1.27	1.44 ± 0.06	1.62	1.42	0.92 ± 0.04	1.63	1.65	2.45 ± 0.18
	*25–44*	0.75	1.10	1.22 ± 0.04	1.31	1.27	1.21 ± 0.07	1.40	1.51	1.53 ± 0.14
	*45–64*	0.76	1.10	1.36 ± 0.08	1.25	1.22	1.15 ± 0.02	0.98	1.12	1.19 ± 0.09
	*> 65*	0.88	1.22	1.35 ± 0.08	1.13	1.19	1.03 ± 0.04	0.64	0.87	0.66 ± 0.08
*Female*	*< 25*	0.89	1.30	2.07 ± 0.18	1.37	1.31	1.30 ± 0.08	1.46	1.60	1.62 ± 0.12
	*25–44*	0.69	1.12	1.80 ± 0.17	1.18	1.11	1.03 ± 0.06	1.14	1.32	1.23 ± 0.08
	*45–64*	0.90	1.26	1.66 ± 0.11	1.19	1.18	1.13 ± 0.04	0.77	1.03	0.84 ± 0.02
	*> 65*	0.69	1.26	1.42 ± 0.09	1.00	1.08	1.13 ± 0.05	0.49	0.74	1.10 ± 0.05

*Frequency:* Values lower than 1 refer to *Frequency* less than twice every 14 days; values from 1 to 2 refer to *Frequency* from once to twice a week; values higher than 2 refer to *Frequency* more than three times a week

*Duration:* Values lower than 1 refer to duration less than 30 min; values from 1 to 2 refer to duration between 30 and 60 min; values higher than 2 refer to duration higher than 60 min

*Intensity:* Values lower than 1 refer to intensity ‘I feel that my body becomes warm’; values from 1 to 2 refer to intensity from ‘I feel that my body becomes warm’ to ‘I feel I breathe harder and get sweaty’; values higher than 2 refer to intensity close to ‘maximum exertion’

Mean of PA *components* class answers from values recorded until 2013 and mean ± standard deviation predicted upon 2025

The low MSA values (i.e., close to zero) and the statistically significant results found in F-test regression analysis indicate that the forecasted evolution of the three PA components predicted by our autoregressive models is highly accurate for both gender in all the age groups.

## Discussions

As previously found by Breivik et al. [8], our analysis shows that Norwegians increased their PA *Frequency* since 1985. Furthermore, the findings of our autoregressive model indicate that this increment is likely to continue up to 2025. While throughout the past three decades PA *Duration* and *Intensity* have not shown noteworthy changes, the autoregressive model indicates that the evolution of these PA components is expected to change. In particular, PA *Duration* is predicted to reduce, while *Intensity* is expected to have a slight increment. With regard to the WHO’s goal of reducing insufficient PA by 10% within 2025, this findings are not encouraging. The expected increase in PA *Frequency* and *Intensity* is in fact likely not to fully compensate the expected decrease in PA *Duration*, resulting in a limited impact in terms of overall PA increments. On the other hand, the predicted increment in PA F*requency* has in itself an intrinsic value, as this can counteract the damages due to prolonged exposure to sedentary behaviour. Sedentary behaviour (i.e. activities such as reading, watching TV, sleeping, etc.) [24] has been more and more seen as an independent factor influencing people’s health. Studies showed that excessive time spent in sedentary behaviour is an independent predictor of cardiovascular diseases, even when individuals meet the minimum PA recommendations [25–28]. This should not however dispense health institutions from engaging in a continuous effort in promoting greater PA engagement in the population, especially by encouraging people to engage in PA for longer durations. Different features were identified as relevant for the different PA components. For example, while PA *Frequency* was associated with giving more value to having a healthy lifestyle, PA *Duration* was rather linked to environmental factors, especially (but not limited to) relative to outdoor PA opportunities and facilities. Furthermore, our analyses also reveal that the predicted evolution of the different PA components is not expected to be equally distributed in the population. This calls for tailored actions targeting specific sub-groups of the population.

A steeper increment of *PA Frequency* was predicted in women compared to men. Moreover, while in the past three decades men appeared to compensate this gap by maintaining higher levels of PA *Duration* and PA *Intensity* than women, this might not be the case in the future: while men are predicted to have greater PA *Intensity* than women by 2025, they are equally expected to have lower PA *Duration*. The WHO’s PA recommendations do state that, when one engages in higher (or vigorous) intensity PA, smaller PA *Duration* is needed in order to gain health-enhancing effects. However, the greater increment in PA *Intensity* predicted for men might not be sufficient to keep pace with women in terms of meeting the PA recommendations, especially when considering the important role of frequent PA in contrasting the negative effects of a sedentary lifestyle. It is also important to notice that the greater increment of PA *Intensity* in men is predominantly driven by the youngest age-group, while men in the oldest age group (who are also expected to have a smaller increment in PA *Frequency* and a larger reduction in PA *Duration*) is expected to reduce the levels of PA *Intensity* by 2025. Thus, these findings suggest that the women of the future might be, overall, more physically active than men. Among women, however, age-related patters must also be noticed. For instance, the magnitude of the predicted increment of PA *Frequency* is expected to be lower in older women than in younger ones.

Overall, the relatively high predictive value of our model confirms the importance of adopting a *social-ecological perspective* in the study (and promotion) of PA in the population [29, 30]. According to social ecological models, health-related behaviours such as PA are influenced by multiple levels of factors (individual, inter-individual, and environmental), which are not under direct control of the individual but can be modified by the society. Quoting Sallis et al. (2006), “multilevel interventions based on ecological models and targeting individuals, social environments, physical environments, and policies must be implemented to achieve population change in physical activity” [29]. Our model proved to be effective in identifying environmental correlates of different PA components, therefore providing useful information not only about the evolution of PA *Frequency*, *Duration*, and *Intensity*, but also about other characteristics of Norwegians PA habits. For instance, according with our analyses, features relative to outdoors PA facilities (especially walking trails) and outdoor PAs (e.g. hiking, cycling, and active transport) were in general relevant predictors of higher levels of the different PA components. Furthermore, in line with previous research [31, 32], features relative to indoor forms of PA and the employment of indoor facilities emerged as relevant in younger age groups. This information is useful in the continuous assurance process of the population health status as well as the evaluation of initiatives aiming to enhance PA in the population.

### Machine learning approaches and its contribution to public health

The technological developments occurred in the past decades have allowed researchers and policy makers to access large volumes of data relative to the health status of the population. This is, for example, the case of routinely administered online surveys. Such sources of data can help researchers to identify and monitor modifiable risk factors associated with non-communicable disease in the population, improving the effectiveness of initiatives to enhance and promote health in the population. This approach to research, however, provides analytical challenges, as the large volume of data could be difficult to manage using ‘classical’ statistical techniques. ML approaches, on the other hand, can be useful tools when dealing with a large volume of data. For example, in the case of this particular study ML allowed us to reduce the set of predictors from the thousands of survey items into a manageable few, and then to use predictive accuracy (that is, the F1 score) to characterize model performance.

It is important, however, to keep in mind the limitations of this technique. For instance, although the Ordinal regression model used in this study showed in general high levels of accuracy compared to RF and the two baselines, the level of accuracy varied across the different PA components, as well as among different age groups (the model was generally more accurate in the oldest age group than in all other age groups). The model also showed higher accuracy for the PA component ‘*Frequency*’, probably due to the fact that this PA component was available for a larger number of survey waves, as compared with the other two PA components. Moreover, higher differences detected between DT and baselines on *Frequency* permits to assert that our algorithm is more accurate in forecasting how often, in average, Norwegians engage in physical activity compared to *Duration* and *Intensity*. Similarly, although the autoregressive model showed high accuracy (as indicated by the MSA values close to zero and the statistically significance found in the F-test), the accuracy of these statistical models could be enhanced by including additional data from further waves of the Norsk Monitor survey. This variations need to be taken into account, and possibly addressed through an inclusion of data of the next years, in order to obtain more valid estimate of PA patterns (or other health outcomes) in different groups of the population.

### Strengths and limitations of the study

Although our analyses are based on the same survey used by Breivik et al. in their study [8], our study provides additional insights. The dataset used in our study was updated with an additional wave of surveys (i.e., we analysed the data recorded between 1985 and 2013, while Breivik et al. used the surveys recorded from 1985 and 2011). Moreover, in their study, Breivik et al. investigate just the *PA Frequency* measurement, while we also examined the measurements of *Duration* and *Intensity*, providing a broader understanding of the extent to which the PA levels in the Norwegian population relate to the national and international recommendations. Moreover, using a ML approach, we were able to perform a more thorough investigation of the patterns and correlates of PA habits in adult Norwegian throughout the past three decades, and we created a predictive model forecasting the future evolution of Norwegians’ PA habits. However, even if the PA component autoregressive models are statistical significant and show a high accuracy, we could only provide a speculation of their evolution because of moderate forecasting uncertain provided as standard deviation in Table 5.

Our study has a number of weaknesses that should be noted. Besides the limitations given by using self-reported assessments of PA, as well as the cross-sectional nature of the study not allowing to establish causal relationships between the different features selected and the respondents’ PA patterns, one of the major limitations of this study is that we could not investigate all the individual characteristics and sociocultural factors recorded through the last three decades by the Norsk Monitor questionnaire because the items have been changed during the years. Finally, because of the way the items were constructed in the Norsk Monitor survey, we were unable to estimate the prevalence of individuals who met (or are predicted to meet) the WHO recommendations for health-enhancing PA. In order to perform better prediction of the extent to which different populations are expected to meet the WHO goals on different health outcomes, it is important that the data collected by national surveys and registers are aligned with the recommendations from national and/or international health institutions.

## Conclusion

This study provides an investigation of the PA patterns and correlates in Norwegian adults over the past three decades, giving a description of the individual characteristics and sociocultural factors that are associated with PA *Frequency*, *Duration* and I*ntensity* in different age groups for both men and women. Using a ML approach, we constructed a predictive model for future PA pattern in adult Norwegians, revealing an expected increment of PA *Intensity* and, especially, *Frequency*, but also a slight reduction of PA *Duration*. Our findings suggest that the overall increment of PA might not meet the WHO’s goal of reducing insufficient PA by 10% within 2025, although some encouraging findings indicate a general reduction of the overexposure to sedentary behaviours. Our findings also bring to light possible disparities between genders and across age groups, calling for tailored interventions targeting in particular men and older individuals. The ML approach used in this study proved to be a valuable tool in performing such investigation, providing useful information to inform initiatives and policies to increase PA levels and reduce sedentary behaviours in specific groups of the Norwegian population.

## Appendix

### The items investigated

In this paper, we use three items as dependent features:

i. *PA Frequency:* How often, in average, do you engage in physical activity in form of structured exercise or general movement? (answers: 1 = ‘Never’; 2= ‘Less than once every 14 days’; 3= ‘Once every 14 days’; 4= ‘Once a week’, 5= ‘Twice a week’; 6 = Three or four times a week’; 7= ‘Five or six times a week’; 8= ‘Once or more times each day’)
ii. *PA Duration:* How much time do you usually spend in each physical activity or exercise session? (answers: 0= ‘Under than 15 minutes’; 1 = ‘15–30 min’; 2 = ‘31–45 min’; 3 = ‘46–60 min’; 4= ‘1-hour to 1-5 hours’; 5= ‘More than 1.5 hours’)
iii. *PA Intensity:* How intense is your exercise/physical activity? Select the answer that fits best you typical exercise/physical activity session. (answers: 0= ‘I don’t feel any change in my breath or body temperature’; 1= ‘I feel that my body becomes warm’; 2= ‘I feel I breathe harder and get sweaty’; 3= ‘I feel I breathe harder, get sweaty, and became tired’; 4= ‘I achieve maximum exertion’)

The alternative answer to these questions were recoded into three levels in order to reduce the answers dispersion and thus increase the robustness of the answers in each question. In particular:

- *PA Frequency*: class 0 refers to answers 1, 2 and 3; class 1 refers to answers 4 and 5; class 2 refers to answers 6, 7 and 8.
- *PA Duration*: class 0 refers to answers 1, and 2; class 1 refers to answers 3 and 4; class 2 refers to answers 5 and 6.
- *PA Intensity*: class 0 refers to answers 1, and 2; class 1 refers to answers 3 and 4; class 2 refers to answers 5 and 6.

### Relevant items emerged through the machine-learning approach

Relevant items selected by RFECV are listed below:

i. To what extent do you agree or disagree with each of the following statement? (answers from 1 ‘Completely disagree’ to 4 ‘Totally agree’)
  1. *Values – Healthy food*: I am more concerned to how good food tastes than to how healthy it is (Q33_1)
  2. *Values – Debts*: It pays to take on debts rather than save up (Q33_8)
  3. *Values* – *Brands name*: I like to wear clothes where the manufacturer’s brand or name is visible
  4. *Values – Marriage*: Being single gives more benefits than being married (Q33_23)
  5. *Values – Personal liberty*: People should be able to look, dress and live as they like, whether or not others approve (Q33_24)
  6. *Values – Children obedience*: The most important thing a child can learn is respect and obedience for their guardians (Q33_25)
  7. *Values – Strikes*: *Strikes* create big problems for society and should be banned (Q33_42)
  8. *Values – Brands quality*: I prefer to buy good quality brands, even if they cost more (Q33_48)
  9. *Values – Gambling*: Forms of gambling like ‘Tipping’, ‘Lotto’ and ‘VikingLotto’ are innocent entertainments (Q33_51)
  10. *Values – Healthy life:* It is important for me to have a healthy lifestyle and to stay in good physical shape (Q26_1)
  11. *Values – Countryside life*: Life on the countryside is more satisfying than life in the city (Q26_3)
  12. *Values* – *Mothers with disabilities*: Physically disabled women (for example, wheelchair users) are equally suit to fill the mother’s role as other women (Q26_18)
ii. *Values – Economic equality*: Do you prefer to increase prosperity in the country or spread prosperity more evenly? (answers form 1 ‘to increase prosperity in the country definitely’ to 5 ‘to spread prosperity more evenly definitely’) (Q27_3)
iii. *View on children number*: What do you think is the ideal number of children for a family in Norway? (answers form 1 ‘No children’ to 6 ‘5 or more’) (Q39)
iv. *Close relationships*: how many of your neighbors have a key to your home? (answers from 1 ‘None’ to 5 ‘a lot’) (Q43_4)
v. *Disagreements with neighbors*: With how many of your neighbors do you have disagreements? (answers from 1 ‘None’ to 5 ‘a lot’) (Q43_5)
vi. *Values – Honesty*: Do you accept to keep money if you get too much in a store? (answers: 1 – ‘Cannot be accepted’; 2 – ‘Can be accepted under doubt’; 3 – ‘Can be accepted’) (Q45_11)
vii. *Views on pension & holidays*: Lower retirement age, reduced working hours, or increased vacation is a big cost for the community. If the costs were the same for 1-h less working time per week, increase of the holiday by 1-week per year, or lowering the retirement age by 1-year, what would you prefer from your situation? (answers: 1 - ‘Reduced working hours’; 2 - ‘Increased holiday’; 3 - ‘Retired retirement age’; 4 - ‘Do not want any changes’) (Q46)
viii. *View on social security*: Many believe that we have received more than enough social security and that we should try to limit it in the future, while others claim that we should maintain our social security systems and, if necessary, enhance it further. What is your opinion? (answers: 1 – ‘Should be reduced’; 2 – ‘Maintained as it is’; 3 - ‘Develop further’) (Q48)
ix. *National pride*: How proud are you being Norwegian? (answers from 1 ‘Very proud’ to 4 ‘No proud’) (Q51)
x. *Language identity*: Do you prefer to re@QWERTYUIOP[21d in Norwegian *Bokmål* or Norwegian *Nynorsk*? (answers: 1 – ‘Norwegian *Bokmål*’; 2 – ‘Norwegian *Nynorsk*’) (Q54)
xi. *Religious identity (Christianity):* Do you consider yourself as a Christian? (answers: 1 – ‘Yes’; 2 – ‘No’)
xii. *Attitudes – State Church*: Are you in favour of the Norwegian State Church? (answers: 1 – ‘In favour’; 2 – ‘Against’) (Q57)
xiii. How well do you think the statements below fit with what you think or do? (answers from 1 - ‘Does not match at all’ to 4 – ‘Very well’)
  1. *Environmental organizations*: I support environmental protection organizations (Q63_2)
  2. *Climate change*: Climate change is largely man-made (Q63_4)
  3. *Religious inquiry*: Religion gives me the best answers to all the essential questions I ask myself (Q63_5)
  4. *Comfort with divergences*: I don’t enjoy the company of people who have different opinions than me (Q63_9)
xiv. *Environmental concerns – Air quality*: How worried are you for the air quality in cities and towns as an environmental problem? (answers from 1 ‘Do not worry’ to 4 ‘Very Concerned’) (Q64_11)
xv. *Environmental behaviors – Active transport*: How often have you use a bike or walk instead of using a car because you wanted to take more care of the environment? (answers from 1 ‘Not Currently’ to 4 ‘Often’) (Q66_5)
xvi. *Childhood in a farm*: Have you grown up on farms? (answers: 1 - ‘yes’; 2 – ‘no’) (Q92)
xvii. Which of the various physical activities listed below do you spend your spare time at least once a month in the appropriate season? (answers: 0 – ‘no’; 1 – ‘yes’)
  1. *PA habit – Jogging* (Q158_1)
  2. *PA habit – Strength exercise* (Q158_9)
  3. *PA habit – Handball* (Q158_17)
  4. *PA habit – Swimming* (Q158_19)
  5. *PA habit – Sailing* (Q158_30)
  6. *PA habit – Cycling* (Q158_33)
  7. *PA habit – Hiking* (Q158_36)
  8. *PA habit – Shooting* (Q158_37)
  9. *PA habit – Motorsport* (Q158_40)
xviii. How often do you engage in sports or physical activity in the following places in the appropriate season? (answers from 1 ‘Never’ to 8 ‘1 or more times per day)
  1. *PA facilities – Sport hall* (Q162_1)
  2. *PA facilities – Track and field stadium* (Q162_5)
  3. *PA facilities – Illuminated track* (Q162_10)
  4. *PA facilities – Walking trail* (Q162_12)
  5. *PA facilities – Outdoor area (Q162_18)*
  6. *PA facilities – Fitness Center* (Q162_19)
xix. There are various reasons for engaging in physical activity and sports. For each of the reasons listed below, please indicate how important they are. (answers from 1 ‘Not important’ to 3 ‘Very important’)
  1. *PA Motive – Health benefits* (Q163_1)
  2. *PA Motive – Challenge* (Q163_3)
  3. *PA Motive – Appearance* (Q163_4)
xx. How important are the following reasons for you not to exercise or exercise less than you would like? (answers from 1 ‘Not important’ to 3 ‘Very important’)
  1. *PA barriers – Lack of time*: I do not have time / it takes too much time (Q165_5)
  2. *PA barriers – Lack of enjoyment*: It is boring (Q165_11)
xxi. *Health beliefs – Snus:* How much of a negative impact do you think ‘Snus’ have on your health? (answer from 1 ‘Very high risk’ to 4 ‘No danger’) (Q284_4)
xxii. *Education field:* What is the major field of study in your education? (answers: 1 – ‘Engineering or other technical subjects’; 2 – ‘crafts’; 3 – ‘law’; 4 – ‘Natural Sciences’; 5 –‘Science’) (Q294)
xxiii. *Work field*: In what field do you work? (14 answer options, e.g. ‘Agriculture / Forestry’, ‘Transport and Communications / Transport / Post / Telecommunication’, and ‘Tourism / restauration’) (Q297)

## Abbreviations

ANOVA: Analysis of Variance
*B*: Baseline
*c*: label
CMIM: Conditional Mutual Information Maximization Method
F: F-score of ANOVA
*F*: matrix
f1: f1-score
*h*: survey answer
m_*i*_: feature vector
Ordinal: Ordinal regression
PA: Physical Activity
Prec: Precision
Rec: Recall
RF: Random Forest classifie
RFECV: Recursive feature elimination with cross-validation
*S*: Feature set
T_*S*_: Training dataset
WHO: World Health Organization
yo: Years old

## Footnotes

1 This was performed using the ***cmim*** function provided by publicly available Python package ***skfeature*** (https://github.com/jundongl/scikit-feature/blob/master/skfeature/function/information_theoretical_based/CMIM.py html/skfeature.html)

2 This was performed using the ***RFECV*** function provided by the publicly available Python package ***scikit-learn*** (http://scikit-learn.org/stable/modules/generated/sklearn.feature_selection.RFECV.html/).

3 We use the ***LogisticAT*** function provided by the publicly available Python package ***mord*** (http://pythonhosted.org/mord/)

4 This was performed using the ***RandomForestClassifier*** function provided by the publicly available Python package ***scikit-learn*** (http://scikit-learn.org/stable/modules/generated/sklearn.ensemble.RandomForestClassifier.html/).

5 This was performed using the ***AR*** function provided by publicly available Python package ***statsmodels*** (http://www.statsmodels.org/devel/generated/statsmodels.tsa.ar_model.AR.html)
